# Procedural pain in children: a qualitative study of caregiver experiences and information needs

**DOI:** 10.1186/s12887-018-1300-y

**Published:** 2018-10-13

**Authors:** Kassi Shave, Samina Ali, Shannon D. Scott, Lisa Hartling

**Affiliations:** 1 Alberta Research Centre for Health Evidence (ARCHE), University of Alberta, ECHA 4-472, 11405-87 Avenue, Edmonton, AB T6G 1C9 Canada; 2 Department of Pediatrics, University of Alberta, ECHA 4-472, 11405-87 Avenue, Edmonton, AB T6G 1C9 Canada; 3 Women and Children’s Health Research Institute, ECHA 4-472, 11405-87 Avenue, Edmonton, AB T6G 1C9 Canada; 4 Faculty of Nursing, University of Alberta, Edmonton, Canada

**Keywords:** Procedural pain, Emergency department, Knowledge translation, Pediatrics, Children, Intravenous, Venipuncture

## Abstract

**Background:**

Children experience multiple painful procedures when being cared for in emergency departments (EDs). Unfortunately, evidence-based interventions to manage such pain and distress are under-utilized across EDs. Caregivers are uniquely positioned and invested to advocate for the adaptation of such evidence into practice. Our objective was to gather information from caregivers of children experiencing procedural pain in the ED to inform the development of a novel, caregiver-focused knowledge translation (KT) tool.

**Methods:**

The study design was qualitative description. Caregivers of children who underwent intravenous (IV) insertion or venipuncture in the pediatric ED at an urban tertiary care centre were interviewed. Thematic analysis was applied to the data. The TRanslating Emergency Knowledge for Kids (TREKK) Parent Advisory Group continuously informed this study, and provided input on interview guide development and piloting, data collection, analysis of the data, interpretation of the results, and development of next steps.

**Results:**

Interviews revealed four major themes: 1) source of healthcare information; 2) delivering healthcare information; 3) communication with caregivers; and 4) procedure-related anxiety and long-term effects. Caregivers most valued receiving information directly from their healthcare provider. They also expressed that healthcare providers should direct information about the procedure to their child and identified strategies to involve children in their care. Caregivers wanted to be empowered to ask informed questions of their healthcare providers. Finally, caregivers reported negative experiences with procedures for their children, occurring mainly at non-pediatric centres.

**Conclusions:**

We have identified core information needs for caregivers whose children are experiencing IV insertion or venipuncture. These results will form the foundation for the development of a KT tool that may empower caregivers to actively participate in their child’s healthcare.

**Electronic supplementary material:**

The online version of this article (10.1186/s12887-018-1300-y) contains supplementary material, which is available to authorized users.

## Background

Pain is a complex phenomenon, and its management is becoming increasingly recognized as the cornerstone of high-quality patient care [1–3]. Optimizing the management of pediatric pain has been highlighted as a key healthcare priority by the World Health Organization, and leading pediatric and pain societies [1, 4–7]. Children experience multiple painful procedures daily when being cared for in hospital and ambulatory settings [8–11]. With half of all emergency department (ED) visits resulting from painful conditions, and 78% of patients experiencing pain during their ED stay [12], EDs represent a setting where effective pediatric pain management should be an essential component of care. The most common painful procedures in the ED include venipuncture and intravenous (IV) insertions [4].

Poorly managed pain from venipuncture and IV insertion procedures can have short-term (e.g., anxiety, avoidance behaviours, and somatic symptoms) and long-term (e.g., increased pain sensitivity, fear, healthcare avoidance as adults) impacts on a child, which can extend and complicate both the procedure and the ED stay [13, 14]. Caregivers may also experience anxiety and distress around their child’s procedure, which, in turn, may increase their child’s perceived pain [15]. There are many evidence-based pharmacological (e.g., topical anesthetic) and psychological (e.g., hypnosis, distraction) interventions available to manage pain and distress in children undergoing procedures [4, 16]. Presently, Canadian healthcare providers are inconsistently utilizing these well-established, effective, readily available, and minimally invasive pain management interventions when caring for children experiencing the most common painful procedures in the ED [17, 18].

Knowledge translation (KT) tools are essential to ensure the uptake of evidence in practice. A number of approaches to translating research evidence into practice have been described in the published literature across various medical conditions and healthcare settings (e.g., clinical practice guidelines, educational interventions) [19–23]. Many of these strategies have been utilized to translate pain management evidence; however, the vast majority targeted healthcare providers outside of the ED context [24–27], and have achieved practice-level change with varying success [28]. Using KT tools to empower patients and their caregivers to play a more active role in their care has been shown to improve health outcomes [29, 30], yet few studies have been conducted focusing on translating procedural pain information for the most common procedures to patients and their caregivers.

The American Academy of Pediatrics and the American Pain Society jointly highlight the need for information to be provided to children and families to ensure they know what to expect when a child is having a procedure and to ensure they are prepared with specific strategies to minimize distress and comfort their children [1, 6]. The first step to engaging caregivers in managing children’s procedural pain is to gather information on their perspectives and needs. The objectives of this study were to determine: 1) what are caregivers’ experiences around painful medical procedures; 2) what are caregivers’ information needs regarding procedural pain and how they can help manage it; and 3) in what format do caregivers want to receive information about procedural pain.

## Methods

A qualitative description approach was utilized in this study [31]. Study recruitment occurred between October 2015 and December 2015 at the Stollery Children’s Hospital (Edmonton, Alberta), a specialized pediatric center whose ED accommodated over 48,500 visits in the 2014–2015 year [32].

Institutional ethics approval was obtained for this study from the University of Alberta Health Research Ethics Board. All potential participants were identified and approached by a physician or nurse on staff when the first author (KS) was present and available for data collection in the ED. KS approached the caregiver to confirm eligibility, invited them to participate in the study, and obtained written informed consent. At the end of the study all caregivers were offered a $10.00 gift card as a token of gratitude.

Caregivers were invited to participate in the study if they had a child between 3 to 12 years of age who had undergone IV insertion or venipuncture in the 4 h prior to recruitment, and self-identified as one of the child’s primary caregivers. Caregivers were excluded from the study if they had a child with urgent medical needs, as determined by the treating ED team, were non-English speaking, or had previously participated in this study. Purposive sampling was utilized to generate an in depth understanding of caregiver experiences [33]. Sample size was determined by saturation of the data, which was monitored through concurrent analysis of the data to assess comprehensiveness, variation, and richness of the interviews [34]. Based on previous research, we anticipated approximately 10–15 interviews would be needed to achieve saturation [35].

One-on-one semi-structured interviews were conducted at the bedside by one interviewer (KS). Caregivers were given the option of having the interview conducted in a private space, but all chose to have their child present. An interview guide (available in Additional file 1) was developed for reference throughout the interviews, and was pilot tested with a sample of research staff and parents prior to participant recruitment. All interviews were audio-recorded and transcribed verbatim. NVivo 10 (QSR International; Melbourne, Australia) qualitative data management software was used for data management during the analysis. Braun & Clarke’s phases of thematic analysis were used [36]. The interviewer (KS) regularly debriefed with the study team. Guba’s naturalistic mode for dealing with questions of trustworthiness was used to guide methodological rigor for this study in terms of credibility, transferability, dependability, and confirmability [37].

All stages of this project were informed by the key stakeholder for this project, Translating Emergency Knowledge for Kids (TREKK), a growing network of researchers, clinicians, national organizations and health consumers who are collectively working to improve emergency care for children across Canada. TREKK collaborates with over 30 general EDs across Canada, spanning 9 provinces and 1 territory. TREKK has a 7-member parent advisory group (PAG) of non-healthcare professionals that includes parents of children with a variety of different health needs and experiences (e.g., acute, chronic, healthy). The TREKK PAG was involved throughout the study, and provided input on interview guide development and piloting, data collection, analysis of the data, interpretation of the results, and development of next steps.

## Results

### I. Caregiver interviews in the ED

In total, 17 caregivers were invited to participate in this study. Of those, 5 caregivers refused consent because they were not interested in participating; 12 caregivers were successfully recruited. Eleven caregivers participated to completion of the interview; 1 interview was interrupted half-way through because their child had urgent medical needs, as defined in the study exclusion criteria. Permission was obtained from this caregiver to use the data collected prior to the point of ending the interview. The rate of participation was 71%. The duration of interviews was between 6 and 34 min, with a mean interview length of 18 min and 24 s.

Demographic variables for all participants (*n* = 12) and their children are presented in Tables 1 and 2, respectively. Analysis of the interviews revealed four major themes: A) source of healthcare information; B) delivering healthcare information; C) communication with caregivers; and D) procedure-related anxiety and long-term effects. See Table 3.

**Table 1:** Caregiver demographic variables

	N (%)
Relation to Child	
Mother	9 (75)
Father	2 (17)
Other	1 (8)
Age	
30–35	5 (42)
36–41	6 (50)
42–57	1 (8)
Marital Status	
Married	9 (75)
Common law	1 (8)
Separated	1 (8)
Single	1 (8)
Highest Level of Education	
High school	2 (17)
Post-secondary	10 (83)
Household Income	
$15–29,000	1 (8)
$45–59,000	1 (8)
$75–90,000	1 (8)
Over $90,000	9 (75)
Ethnicity	
Caucasian	11 (92)
Other	1 (8)

**Table 2:** Child demographic variables

	N (%)
Age	
3–6	4 (33)
7–9	4 (33)
10–12	4 (33)
Procedure	
Intravenous	11 (92)
Venipuncture	1 (8)
Medical History	
Healthy	2 (17)
Chronic illness	10 (66)
Prior ED Visits	
0–4	6 (50)
5–9	1 (8)
≥10	5 (42)
Admissions to Hospital	
0	4 (33)
1	5 (42)
> 1	3 (25)
Long Term Admissions (> 45 days)	1 (8)
ICU Admissions	2 (17)
# of Intravenous Insertions (*n* = 11)	
1st time	2 (18)
2–5	6 (55)
> 20	3 (27)
# of Venipuncture Procedures (*n* = 1)	
> 10	1 (100)

**Table 3:** Description of themes and subthemes with example quotations for caregiver interviews in the ED

Theme and sub-themes	Example of statements
A. Source of healthcare information	
Videos are a neutral medium for sharing information	*“I think hearing it from the nurse is fine, if the child feels comfortable with that person. But you don’t really have a way of gauging that, necessarily, every time, depending on how much pain they’re in, or why they’re here. If they’re physically really, really sick, I think a video would be cool”* [mother, 5 year-old girl]*.*
Videos as a tool for distraction	*“[The video] can kind of distract them as they’re watching it. Even if they could be watching a video that explains what’s going to happen, in a really friendly, easy, kiddy kind of way, you know, and then, even if it could go on a little further… and then kept it going while [the procedure] was happening… I think that would help. Because then they’d still be distracted”* [mother, 5 year-old girl].
Posters stimulate conversation with healthcare providers	*“For an older child this age, probably a poster would work. That way, if you had to have the visual, then you’d think “oh, maybe I should ask about this”*” [mother, 12 year-old girl].
Pamphlets can be referred to in the future	“*I don’t throw [the brochures] away. I keep them in case somebody else gets hurt – in the same situation to see how I can manage them, to help with them”* [mother, 3 year-old boy]. “*I am old school. I like paper. I can refer back to it. And have it in front of me”* [mother, 9 year-old boy].
B. Delivering healthcare information	
Treat the child as someone who is going to understand	*“Today [my daughter] was very calm, and everything was explained to her. She was treated like someone who’s going to understand, instead of just a kid and you know, you’re – you’re being out of control or anxious, and let’s just get this done, so… the patience, the compassion, everything that has been said is really important”* [mother, 5 year-old girl]..
Ensure the child knows what to expect	*“[My daughter is] at the age where she wants things explained to her. The more information she has, the better [the procedure goes]. So it’s better for the doctor or the nurse, just to talk directly to her, at this age”* [mother, 12 year-old girl].
Describe the procedure in a way they understand and can relate to (e.g., use “kid terms”)	“*[The nurse] was explaining what she was looking for and described the veins to him in kid terms”* [mother, 8 year-old boy]. Another mother elaborated on this concept by providing an example of how “kid terms” were used when her daughter had an IV insertion: *“[When] they explained the freezing gel and how it’s supposed to make her feel, they compared it to Elsa and how it’s going to be frozen and, of course, every kid loves Elsa right now, so that helped too – relating it to things that kids know…”* [mother, 5 year-old girl]
Give the child adequate processing time to understand and prepare themselves for the procedure	*“[My daughter] needs to know. She needs time to process what’s happening. And then, when she – [the nurses] were more patient with her, to – then she watches them put the needle in. Which seems to help her. So today was good”* [mother, 12 year-old daughter].
Involve children in the procedure by giving choices or opportunities for decision-making or other input where possible	*“[The nurse] was very calm in asking [my daughter] how she wanted [the IV procedure] done. So it was more so letting her know that this isn’t being done to you – that you’re still a part of this. And just letting her know… okay, do you want the board underneath your arm? Or, “does it still hurt?” And just letting her know what’s gonna happen next. You know what I mean? So I think… just strategically telling her about [the procedure], and calmly doing it in a manner that is not going to scare her.”* [mother, 5 year-old girl]. *“I do like that they explained to him and involved him in what’s going on. It prepares him, right?”* [mother, 9 year-old boy].
C. Communication with caregivers	
The need for reassurance	*“[During the procedure] have the doctor say, “No, you’re doing good, dad. Just keep talking to [your son], keep talking to him.” So maybe, if that’s explained ahead of time, that you should do what naturally comes to you, like for distraction just talking to him, have him look at you in the eyes. Ah, that – feel free to do that. And if there’s something that you shouldn’t be doing or that may obstruct the medical procedure, [the doctor] will let you know. But otherwise, continue what you’re doing until you’re told otherwise”* [father, 7 year-old boy].
Information about Maxilene	*“I’m not sure too much about the information about the cream. So maybe… information about that would help me out. Yeah. ‘Cause they don’t normally give it to [my son] at the bloodwork clinic”* [father, 6 year-old boy].
Information about the nature and duration of pain	*“Is it gonna hurt right now? What’s it gonna hurt? How long is it gonna hurt for? Ah, would be good to know beforehand”* [mother, 12 year-old girl].
More information about potential discomfort after the IV has been inserted	*“I think, more for the child to be told… that not only will there be an initial pinch – but it will probably be uncomfortable… for the duration that it’s in. So that they don’t think once the pinch is done, it’s all done. But rather, that if someone pulls on it or you turn the wrong way, there’s still going to be that discomfort that happens”* [mother, 8 year-old boy].
D. Procedure-related anxiety and long-term effects	
Increasing comfort with procedures as child gets older	*“Now it’s okay. ‘Cause I know that [my son] is going to be okay. When he was younger, we’d be a bit nervous, ‘cause you never know how they’re going to react [to the procedure].”* [aunt, 10 year-old boy].
Strong preference to have their child’s procedure done in a pediatric centre	“*People, I think [at the Stollery Children’s Hospital] people are… people are more aware of how to treat a child. Whereas outside, sometimes… they’re not as… caring…”* [mother, 4 year-old boy].

### A. Source of healthcare information

Caregivers discussed multiple sources through which information about IV insertions and/or venipuncture procedures could be provided, including from their healthcare providers, videos, posters, and pamphlets (Table 3). Overall, caregivers most valued receiving information directly from their healthcare provider. Caregivers valued the efforts of their nurses and/or doctors when they explained the procedure to both themselves and their child. Further, they appreciated ongoing dialogue and explanation during the procedure. One caregiver stated: *“As a parent, [having the procedure described] by the nurse, step by step, is great”* [mother, 8 year-old boy]. Another highlighted the importance of the doctor explaining the procedure to her child: *“I did find it quite nice that the doctor was the one that [explained the procedure]. ‘Cause I think sometimes that helps more for kids – that authority…”* [mother, 8 year-old girl].

### B. Delivering healthcare information

Caregivers discussed who should be targeted when sharing information, and how that information should be shared. Caregivers emphasized the importance of keeping procedure-related information focused on the child. One caregiver said:

*“I’m used to [the procedure]. I’m used to seeing her get IVs, I’m used to seeing her get needles. I give her needles every day. So for me, it’s okay. I just want to make sure that she’s calm enough that she’s not going to move while they’re doing what they need to do, or that she understands that she’s safe. That it’s okay, no one’s leaving. That she’s not alone, so um… And she’s alright with that, as long as she knows that it’s going to be quick. And people are just taking their time with her and explaining steps to her. Then she’s better with it”* [mother, 5 year-old girl].

Caregivers also highlighted the importance of their child being actively involved in their own care. The following components and/or elements were identified to best involve children in their care: 1) treating children as someone who is going to understand; 2) ensuring children know what to expect; 3) describing the procedure using language and concepts that are familiar to children (i.e., ‘kids terms’); 4) giving children processing time; and 5) involving children in the procedure (Table 3).

### C. Communication with caregivers

Caregivers emphasized the importance of open communication with their healthcare providers. Focusing on empowering caregivers to communicate and providing reassurance were highlighted as important to best facilitate communication between caregivers and their child’s healthcare provider.

Caregivers expressed that receiving procedure specific information through various sources would have benefits beyond gaining access to educational materials, as it might facilitate communication with their healthcare providers. One caregiver discussed the potential of posters to spark conversation: *“I think the poster would be good at this point in time for my age kids. I really do. It’s visual. That way it would spark conversation. It would be good”* [mother, 12 year-old girl]. Another suggested the potential for a video to inspire questions that he can then later bring up when his child’s physician is explaining the procedure in detail:

*“… And then, when you sit down with your anesthesiologist, and he’s gonna explain the IV. Then, if you have questions, you could say, okay, when he’s doing this, what – you know, I read – I saw this little video. Is this okay if I do this, if they have any questions”* [father, 7 year-old boy].

Needing reassurance from the healthcare provider throughout their child’s procedure was also brought up as a meaningful way to support caregivers (Table 3).

Throughout the interviews, caregivers identified specific areas where more information needed to be communicated regarding their child’s procedure. First, some caregivers felt they or their child was lacking basic information about Maxilene™. Maxilene, a fast-acting topical anesthetic lidocaine cream, was offered to all children in this sample; all but one accepted it. Although caregivers nearly unanimously (*n* = 11/12) agreed that Maxilene was helpful for their child during the procedure and that they would request it again for future procedures, some caregivers felt they or their child lacked basic information about the cream (e.g., what it is, how it works, how long it lasts). Second, caregivers expressed the need for information about the nature and duration of pain prior to the procedure (Table 3). Finally, while caregivers felt that the immediate procedure itself was generally well described, one caregiver thought their child could have received more information about potential discomfort *after* their IV had been inserted.

### D. Procedure-related anxiety and long-term effects

Caregivers shared many stories of their child’s prior experiences of having painful procedures. Although each experience was unique, caregivers consistently reported the enduring negative impact of their child’s experiences. One mother reflected on her daughter’s fear of having an IV insertion:

*“She was crying [today during the procedure]. Just the fear, again. Because she’s done this so many times and she knows… And I think, really, the first time that she ever experienced it, when she was originally diagnosed with Type 1, they had to use her feet – so they were poking her feet, they were poking her arms, they were poking her fingers, they were poking every inch of her. And that stays with her. She’s… the kind of kid where, something happens, it’s almost traumatic to her. And – and she never forgets”* [mother, 5 year-old girl].

Procedures in young children were emphasized to be the most challenging, stressful procedures for caregivers. One father shared: *“At the beginning it was real tough. To hold [my son], and he’d kinda fight ya – and it almost felt like you were gonna break him, almost”* [father, 6 year-old boy]. However, caregivers expressed a greater sense of comfort with procedures as their child became older and more experienced with procedures.

Caregivers reported strongly preferring to have their child’s procedures done in a specialized pediatric centre. One mother stated:

*“In [rural town] this morning, they flat-out said that they’re not used to working on kids. So – ‘cause they tried to put an IV in him there and they couldn’t get it in. So… it’s just different here. The people here at the Stollery work with kids, so it’s – just a little more comforting knowing that, too. That… they’re used to working with the little veins…”* [mother, 4 year-old boy].

Another mother drew on her experiences at an urban phlebotomy clinic: *“They’re more… harsh at [urban phlebotomy clinic]. They don’t really explain what they’re doing. They just sit you down and… take blood out of you and away you go.”* [mother, 12 year-old girl].

Caregivers also shared concerns about their anxiety. These concerns were often centered on complications arising from their child’s procedure and their child’s anticipation of the procedure. One caregiver stated: “*Well, there’s always so many – I don’t know, I always worry if he jerks the wrong way, if – they’re gonna cut him, or something. I don’t exactly know what they do even, so…”* [mother, 4 year-old boy]. Caregivers also expressed concern about their child requiring multiple needles: *“I was concerned if she was actually gonna hold still, and if she was gonna have to get poked again.”* [mother, 10 year-old girl]. One mother emphasized the importance of addressing caregiver anxiety, she said: *“Sometimes, maybe the parents might need more support than the kid. ‘Cause [my daughter] was okay [when she had her procedure], and I was in tears”* [mother, 12 year-old girl].

Caregivers also shared concern about their child’s anxious anticipation of the procedure. Specifically, caregivers reported their child’s anxiety to be the worst before the procedure. One caregiver stated: “*I think it was more the before the IV part that was a problem, because he was anxious about the idea of getting one more than the actual reality of one”* [mother, 8 year-old boy].

### II. TREKK parent advisory group discussion

Overall, members of the TREKK PAG felt that the experiences of caregivers in this study were in keeping with their own. The caregivers also expressed the potential for these results to have applicability in both pediatric and adult healthcare settings (see Table 4). However, the TREKK PAG highlighted the differences in care children receive in general and rural healthcare settings, and the necessity of this being further explored to facilitate KT that is relevant and accessible to these environments.

**Table 4:** Description of themes and subthemes with example quotations for TREKK Parent Advisory Group discussion

Theme and sub-themes	Example of statements
Applicability in pediatric and adult healthcare settings	*“Parents are dealing with [communication challenges] in any situation, whether we are at our doctor’s or we’re at the ED or whether we are at the Children’s ED...”* [P5].
Differences in care received in general and rural healthcare settings	*“The doctor actually told me that we shouldn’t have come [to a general ED], because they’re just not trained. And then, if a kid was actually really sick, that they would just send him by ambulance to the Children’s Hospital. That was eye opening”* [P2]
KT tools facilitate communication with healthcare providers	*“In my experience, once we established ourselves as being credible [parents], that we’re trying to be part of the solution here and not be part of the problem – because quite a few times you can come off as being a little pushy or whatever – but once we establish that we are here to help. I’m not trying to tell you how to do your job or anything like that. Once it gets past that barrier, then I find you get better communication. A lot of things go a lot smoother, just because you’ve established that credibility” [P2].*
KT tool needs to be mindful of time and resource constraints in EDs	*“…There would have to be options, but not so many options that we’re overwhelming the nurses. Because, I mean let’s face it. How many questions are these men and women getting anyway? Let’s not overwhelm their jobs… I want to see both sides with some sort of happy medium, if that’s possible. ‘Cause you know, let’s not piss off the industry, but let’s help the parents”* [P5].
KT tool needs to be available in more than one format	*“It’s audience as well, right? You have to look at demographics too, and who’s bringing the people in. So, when you’re looking at anybody below the age of – I don’t know, my mom’s 75, and she’s on the smart phone all the time. But that could be a simple question that’s asked, right? Like, “what’s your preference?””* [P6].
Education should be provided during a waiting period	*“The reality is, you’re gonna be waiting [in the ED]. That’s a given. So give people something to make that wait a little bit more useful”* [P2]. *“And at that time, they could be educating their kids and themselves”* [P3].
Waiting period gives caregivers time to think about what they have learned and prepare questions	*“Then [parents are] thinking, you’re going to be asked [about the procedure]. And now you’ve got time to think about it”* [P1].
Procedural pain menu	*“It might be good to have some sort of… cue, or something that I could think about what techniques would work for my particular child who is there. I mean, you have, usually, a long time to wait when you go. So it might be a good time to spend that time coming up with things that would be particularly helpful for my children… And maybe spending that time while you’re waiting with a menus or checking off what would work for your kid might be helpful to communicate to the healthcare providers what your particular child might want”* [P6].
Colouring book	*“In the kid setting, how hard would it be to come up with, say, a colouring book of someone going to get their needle or an IV put in. And then maybe a few lines on each page describing what will happen, which the parents can go through with their child to maybe have them think of some questions that they will need [to ask]…”* [P3].
Mobile application	*“What about an app that can be created, in terms of those pieces that you could quickly call up – or when you’re checking in, that you could fill in, in some sort of a form. Because that’s kind of what we use on a daily basis”* [P1]. *“I like that idea. Because not only could you use that, it would be easy to transfer to any facility, right?”* [P6].
Comprehensive digital platform that can be accessed via hospital Wi-Fi	*“When you register in the ER, they should be directing you to use the Wi-Fi. At the front page of your Wi-Fi, you should have these links to different [resources]…”* [P2].

The TREKK PAG highlighted a number of benefits to developing a KT tool for caregivers of children undergoing IV or venipuncture procedures in the ED setting (Table 4). They suggested caregivers may have questions that they don’t know how to ask, when their child is having a procedure. Primarily, KT tools were discussed as a valuable strategy to facilitate communication with their child’s healthcare provider.

Based on the interviews, the TREKK PAG suggested a number of key components to be considered when developing a KT tool on procedural pain management (Table 4). First, the TREKK PAG suggested developing a tool that is mindful of the time and resource constraints in EDs. They also suggested having KT tools available in more than one format (e.g., video and brochure) to accommodate different audiences and preferences. The TREKK PAG suggested that it would be beneficial to receive education during a waiting period because it gives caregivers time to think about what they have learned and prepare questions for their healthcare provider.

A number of ideas for specific tools were discussed among the TREKK PAG (Table 4). The first tool suggested was a procedural pain menu that would allow caregivers to see what is available within their ED, and check off what they would like to be made available for their child. The second tool suggested was a colouring book that followed the story of a child going to have an IV insertion. A colouring book was seen as particularly beneficial because it is something that could intentionally engage both caregiver and child. Finally, the TREKK PAG suggested the development of a mobile application, specifically built as a digitized format of the procedural pain management menu. A mobile application was seen as a potentially useful tool specifically because it is accessible on devices familiar to caregivers and children, and can be made widely accessible across hospital sites. One father built on the idea of a mobile application, and suggested the application as becoming part of a more comprehensive platform that incorporates information on a variety of common hospital experiences and procedures, and could be accessed when connecting to hospital Wi-Fi.

## Discussion

This study demonstrates that while many positive steps are being taken to manage children’s pain and provide both children and their caregivers with information prior to and during IV insertion or venipuncture in the ED, greater efforts are needed to empower children and their caregivers to have a more active role in their healthcare across all clinical settings. Further, there are likely differences in the management of procedural pain in children across different types of sites (e.g., general EDs, rural EDs, non-pediatric phlebotomy labs).

These results show that considerable efforts are being made at our study site (i.e., pediatric ED) to implement evidence-based strategies for pharmacological management of procedural pain. Ensuring children are presented with the option of having a topical anesthetic (Maxilene) applied prior to painful procedures, such as IV insertions and venipuncture, is a departmental priority. In our study, all caregivers reported that their child was offered Maxilene. All caregivers of children who utilized Maxilene were pleased that their child was given the option of having the anesthetic applied, thought it helped their child, and would request it again for future procedures. Nurse-initiated protocols exist within this site for a number of pain management strategies including acetaminophen, ibuprofen, and Maxilene application, establishing the department as one of few Canadian pediatric EDs with pain-specific nurse-initiated protocols in place [17]. Empowering nurses to provide pharmacological pain management techniques not only reduces the pain children experience, but also decreases the time to analgesia and increases the number of children who receive analgesia in the ED [38]. Having a Maxilene nurse-initiated protocol in our study site very likely positively impacted the experience of caregivers and children included in this study.

All of the caregivers in this study described being pleased with the care their child had received in the ED. Throughout the interviews, caregivers emphasized the value of receiving information about the IV insertion or venipuncture procedure directly from their healthcare. The majority of caregivers described feeling well informed about their child’s procedure, however, expressed that the issue of greatest importance was ensuring their child felt well prepared and supported throughout their procedure. Caregivers suggested healthcare providers should treat children as someone who is going to understand, ensure children know what to expect, describe the procedure using language and concepts that are familiar to children, and allow children to make decisions or choices where possible, as effective strategies for supporting and involving children in their care [39].

The experiences shared by the participants support the findings of published research which indicate providing analgesia, alone, does not predict patient and caregiver satisfaction with ED care [40]. It was been well established that effective communication between patients, caregivers, and their healthcare provider leads to greater self-reported patient and caregiver satisfaction [40, 41]. Effective communication strategies, showing kindness and patience, speaking directly with a child, incorporating the child’s knowledge and preferences into a procedure, and providing developmentally appropriate information and education using language that is familiar to the child, are essential to providing family-centred pediatric care [39]. Caregivers and patients who feel heard, are involved in decisions about their care, and receive appropriate information report less anxiety and interpret their experience more positively when being cared for in the pediatric ED context [39]. Given the importance of communication, the methods for communicating healthcare information described by caregivers in our sample, while intended for children in the ED, would have utility across all healthcare settings.

By providing caregivers and children with information about procedures, the caregivers in our sample described potential opportunities for education, stimulating conversation about the procedure, and giving caregivers the information they need to ask informed questions to their healthcare providers. Ongoing Canadian initiatives such as Be Sweet to Babies [42], the Commitment to Comfort Program [43], and It Doesn’t Have to Hurt [44] are investigating the impact of KT tools targeting caregivers, specifically, videos, posters, and social media, on the management of pain across various healthcare settings, and for different populations and indications. Using KT tools to empower patients and/or caregivers to play a more active role in their care has been shown to improve health outcomes [29, 30], yet to the best of our knowledge, no studies developing or testing caregiver-focused KT tools for pediatric procedural pain in the ED setting have been conducted.

Participants frequently described anxiety around procedure-related complications (e.g., their child being cut) and their child experiencing greater pain from multiple needle pokes due to unsuccessful procedures. Effectively utilizing analgesia for procedures such as IV insertions and venipuncture can not only substantially improve the pain children experience, it can also improve the success rate of procedures and shorten the overall procedure time, highlighting, again, the importance of effective knowledge translation in this area [45]. Caregivers shared distressing experiences about procedures carried out at non-pediatric urban and rural centres. In a recent survey of pediatric pain management practice and policies in Alberta EDs, it was noted that policies around procedural pain were lacking [18]. Implementing pain-related policies has been linked with an increase in the number of patients receiving analgesia for procedures in the ED setting [46]. Given 85% of Canadian children in need of emergency care are seen in non-pediatric EDs, there is an urgent need for KT efforts targeting the general ED setting [47, 48].

While our participants provided in-depth descriptions of their experiences and information needs when their child is undergoing a painful procedure in a pediatric ED, this study is limited by a number of factors. Our sample was limited to primarily Caucasian, married females, who have a post-secondary education and report a household income greater than $90,000 per annum. Further research is needed to understand the experiences and information needs with consideration of broader ethnic diversity, educational background, and socio-economic status. Our sample also primarily included children who have chronic illnesses, and as a result, several had previous experience with painful procedures. Caregivers of children who are having first time procedures may have different experiences and information needs than more experienced caregivers of children who have had multiple painful procedures. Similarly, most of the procedures (*n* = 11) experienced by the children of caregivers in our study were intravenous cannulation. Given the distinct nature of venipuncture, caregivers of children having these procedures may have different experiences and information needs that were not reflected in this study. Further research is required to better understand the experiences and information needs of these caregivers. Additionally, because this study was conducted in an urban pediatric centre, the results may not represent the experiences of caregivers and children undergoing painful procedures in other contexts (e.g., rural ED). Data collection also occurred in the ED, with the child present. This environment, as well as their child’s presence, may have impacted the caregivers’ responses and how they shared their opinions about their child’s procedure. Finally, our study is limited by caregivers’ previous experiences and knowledge regarding painful procedures and the minimizing of associated pain; if they were unaware of available pain treatment modalities that could have been offered to them, they would not be in a position to report dissatisfaction with not receiving them. The impact and relevance of distraction techniques were not explicitly described in the interviews with caregivers.

## Conclusions

KT tools targeting caregivers are an important and potentially effective way of sharing research evidence in a form that is accessible and catalyzes change in practice. Caregivers in our study report that poorly managed procedural pain continues to persist in the emergency care setting, and we know from previously published research that this may cause long term negative effects for both caregivers and children. Caregivers want to better understand how they can reduce their child’s pain, and they want to learn from and work together with their healthcare providers to manage their child’s pain. Empowering caregivers to ask informed questions and request evidence-based pharmacological and non-pharmacological treatments is an important next step in closing the knowledge to practice gap in pediatric procedural pain management.

## Additional file

Additional file 1: Interview Guide. (DOCX 13 kb)

## Abbreviations

ED: Emergency department
IV: Intravenous
KT: Knowledge translation
PAG: Parent advisory group
TREKK: TRanslating Emergency Knowledge for Kids
