# Supplementary material for: Procedural pain in children: a qualitative study of caregiver experiences and information needs
Source: BMC Pediatr. 2018 Oct 13;18:324. doi: 10.1186/s12887-018-1300-y (PMC6186099; doi:10.1186/s12887-018-1300-y)
Supplement: Supplementary file 1 — Interview Guide. (DOCX 13 kb) [file 12887_2018_1300_MOESM1_ESM.docx]

**Additional File 1. Interview Guide**

**Procedure**

1. Tell me about your experiences when your child had the bloodwork/IV insertion today.
2. What was it like for you as a parent when your child had the procedure?
3. What strategies were put in place by healthcare professionals to help your child?
4. Did you do anything to manage your child’s pain during the procedure (e.g., distraction or incentives)?
5. How did your child cope with the procedure?
6. What were your child’s emotions like before/during/after the procedure?
7. What were your emotions like before/during/after the procedure?
8. Has your child ever had a painful procedure done outside of the Stollery? If yes, how would your experience there compare to here?

**Information**

1. How well was the procedure explained to you prior to your child having bloodwork/IV insertion?
2. What information would you have liked to receive so you could help your child through the procedure today?
3. How would you have liked to receive this information?
4. How would you seek healthcare information if you were at home, your child was sick, and you didn’t have easy access to a health care provider?
5. What makes you trust healthcare information?
6. If there were new effective ways to manage your child’s pain that you didn’t know about, and your health care provider didn’t immediately explain to you, how would you like to receive information about these pain management strategies?

**Other**

1. Do you have any advice for parents of children having bloodwork/IV insertion?
2. Would you do anything differently if your child needs bloodwork/IV insertion in the future?
3. Do you have any stories you would like to share with me?
